# Promoting equity for a better quality of care for all Europeans

**DOI:** 10.1080/13814788.2018.1530506

**Published:** 2018-10-30

**Authors:** Adam Windak

**Affiliations:** Department of Family Medicine, Jagiellonian University Medical College, Krakow, Poland


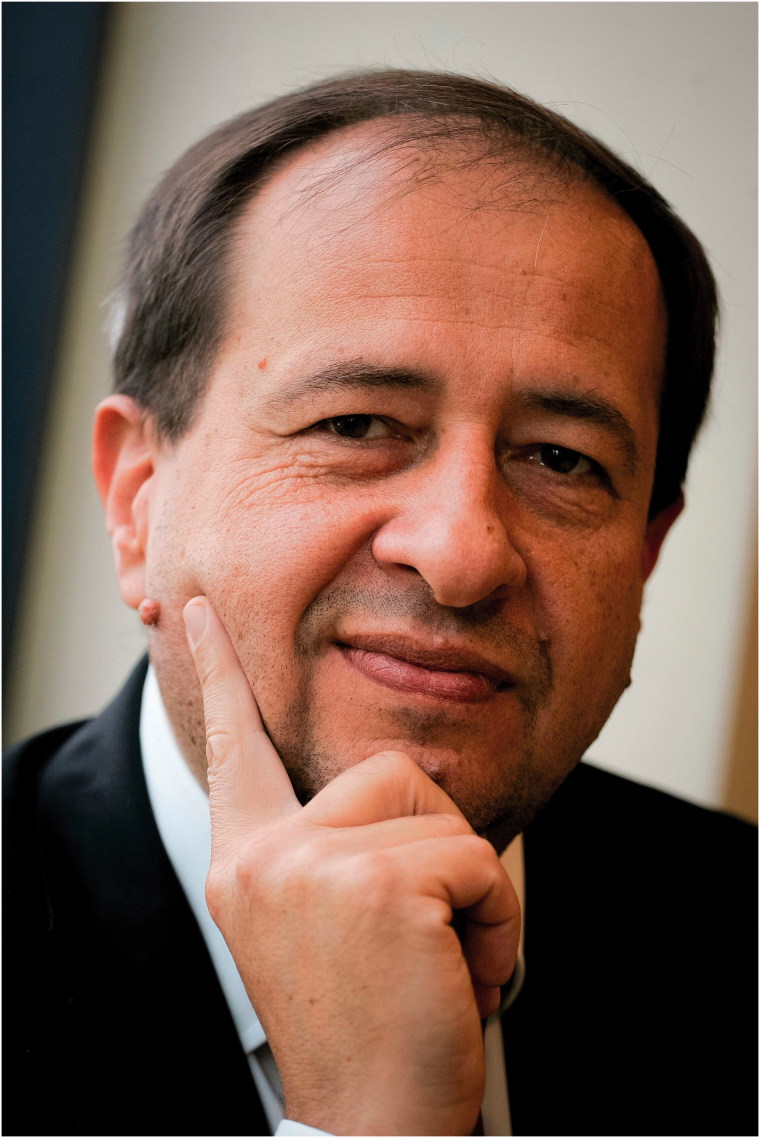


At the end of May 2018, the 23rd Wonca Europe Conference was held in Krakow, Poland. Its theme was ‘Family medicine: quality, efficiency, equity.’ These three aspects correspond closely with the core values of our discipline. Why do we focus on them now in 2018? Is the delivery of quality, efficiency or equity by family physicians in Europe under threat? If so, by which factors or phenomena?

One factor that comes to our minds is undoubtedly migration. According to the Eurostat data in 2016, over two million immigrants arrived in the European Union from other countries [1]. They came here escaping from wars and persecution in their home countries or merely wanting to secure a better future for themselves and their children in stable and wealthy Europe. According to the same data source, there are now 22 million non-EU citizens living in 28 EU countries. This means that almost 5% of the European population is made up of immigrants. Most of them may have poorer access to medical services than other Europeans due to various factors, e.g., economic, linguistic, cultural or religious. Countries like Germany, Belgium or France, which were the traditional destination of immigrants for several decades, have managed to create social support, cultural and practical solutions, including medical coverage. For many others, this is a new situation, which requires special attention, resources and organizational changes to meet these challenges.

The participants of the Wonca Europe Conference in Krakow addressed these issues in the statement with the same title as this editorial [2]. They called for the action of politicians to secure equal access to healthcare for all inhabitants of Europe and urged their colleagues to be aware of the importance of equity and interprofessional collaboration to achieve it. They also stressed the need for action by Wonca Europe Member organizations to collaborate with governments and other policy and decision makers to ensure equal care for all. Finally, they underlined the necessity of alterations in medical educational systems to enable the family medicine workforce to meet the health needs of all people living in Europe. In this respect, the Krakow declaration is in line with, and reinforces, the one announced in 2015 during the Wonca Europe Conference in Istanbul. That document focused on the refugee crisis, which indeed is not over yet [3].

How can individuals, organizations or governments respond to this call? First of all, doctors should practice according to the principles described in the European definition of our discipline, which says that general practice ‘… is normally the point of first medical contact within the healthcare system, providing open and unlimited access … regardless of the age, sex, or any other characteristic of the person concerned …’ [4]. That is a moral and ethical obligation for all European GPs.

What can organizations do? There are several local initiatives, but an interesting example of international collaboration in this field might be an EU-funded project titled ‘Provision of training for first-line health professionals and law enforcement officers working at the local level with migrants and refugees’ [5]. The project, conducted by an international consortium led by the United Nations International Organization for Migration, will develop courses for primary healthcare clinicians and others aimed at reinforcing the skills required promoting understanding, positive attitudes, and a holistic approach in work with migrants and refugees. The European Academy of Teachers in General Practice/Family Medicine (EURACT) has agreed to collaborate with the consortium to enrol the best potential participants in the project activities.

Equity in healthcare is also a challenge for researchers seeking the best measure for it. A recent systematic review of the studies in the field had major difficulties finding a universal measure and concluded that further research is needed [6]. Razum et al. presented an interesting concept arguing that the uptake of antenatal care might be a good indicator with which to measure potential inequities [7]. Further research on the subject should be of interest not only for scientists and policymakers but also for GPs dealing with the problem in their practices.

How to provide adequate and equal primary care to all Europeans, including the deprived populations of immigrants, is a difficult question to answer. On 21 – 22 September 2018, the 2nd EURACT Medical Education Conference was held in Leuven, Belgium. During one of its workshops, participants discussed the issues of social and health inequities with Jan De Maeseneer, who recently wrote a book summarizing his 40 years of experience as a GP, scientist, and healthcare expert [8]. According to him, immigrants should be entitled to regular healthcare services like other Europeans and general practice/family medicine is the best place to secure it. There is no need for special services. ‘Care designed just for the poor generally is poor care,’ he said. That is difficult to deny.
